# Supplementary material for: Pharmacological and Genetic Disruption of C-Type Natriuretic Peptide (nppcl) Expression in Zebrafish (Danio rerio) Causes Stunted Growth during Development
Source: Int J Mol Sci. 2023 Aug 18;24(16):12921. doi: 10.3390/ijms241612921 (PMC10454581; doi:10.3390/ijms241612921)
Supplement: Supplementary file 1 [file ijms-24-12921-s001.zip › ijms-2552882-supplementary.pdf]

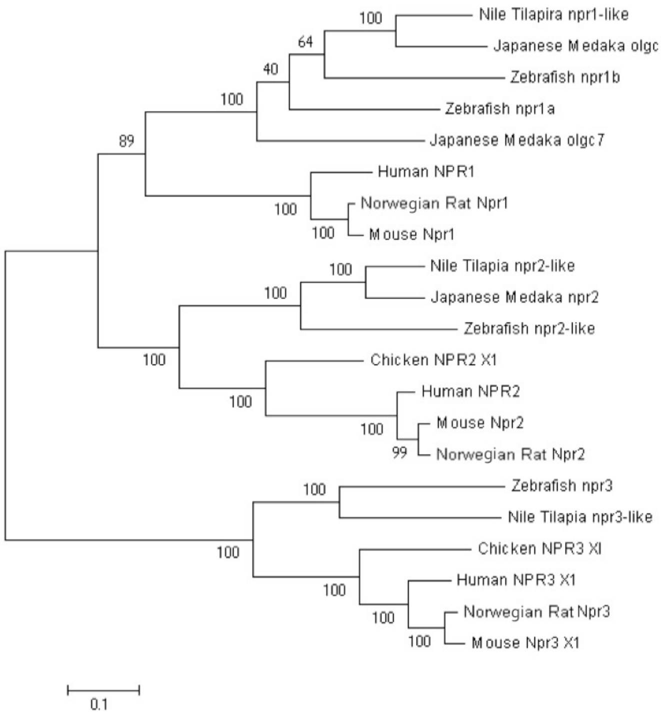

Supplemental Figure S1 Phylogenetic tree of natriuretic peptide receptors including NPR1 (GC,A), NPR2 (GC-B) and NPR3 To examine the evolutionary relationships of zebrafish natriuretic peptide receptors, multiple sequence alignments of published whole natriuretic peptide receptor sequences were aligned using Clustal Omega (http://www.ebi.ac.uk/Tools/msa/clustalo). Evolutionary genetic analysis was performed on the resulting alignment using MEGA5 (http://megasoftware.net) and a phylogenetic tree created using the maximum-likelihood method and Tamura-Nei substitution model. Nonparametric bootstrap sampling was performed (1000 replicates) alongside and shown as a percentage at each corresponding node. Scale bar represents a genetic distance of 0.1.

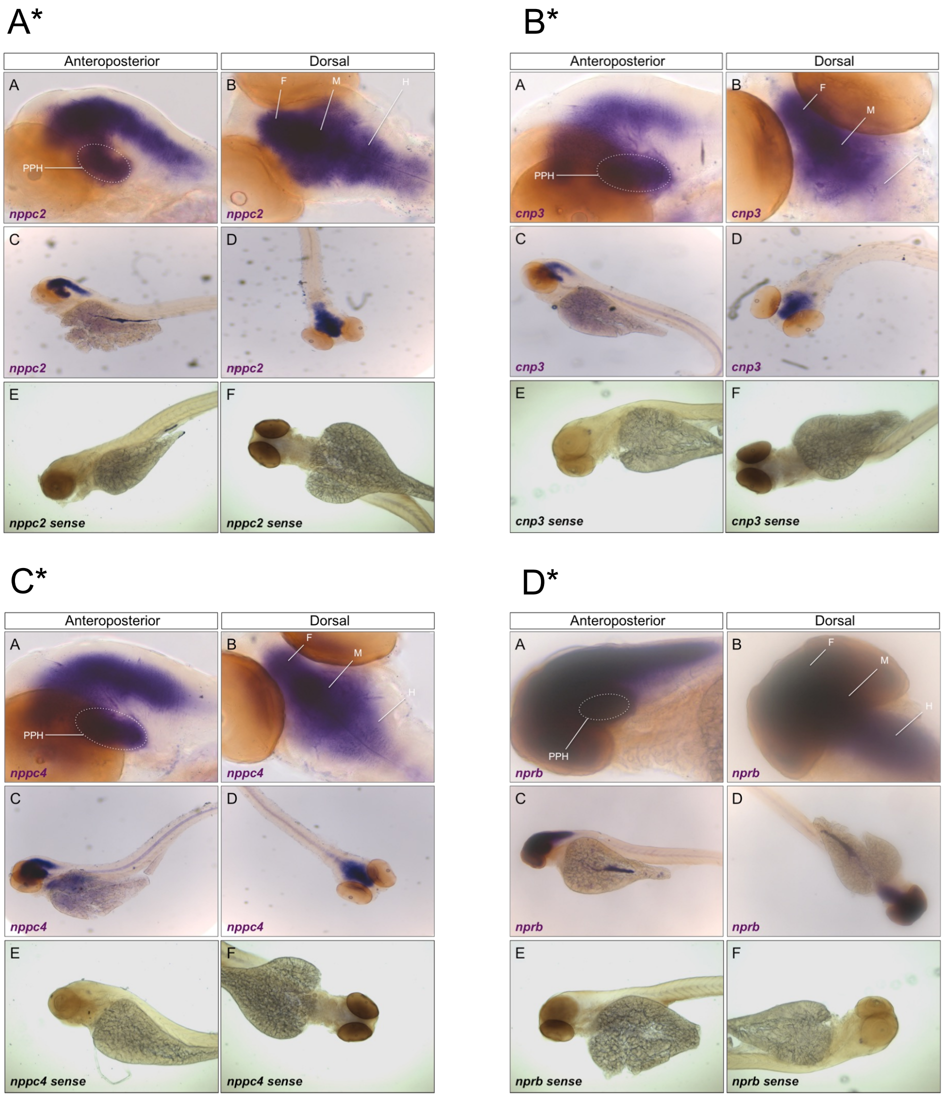

Supplemental Figure S2A-D* Wholemount in situ hybridization of (**A***) *nppc2*, (**B***) *cnp3*, (**C***) *nppc4* and (**D***) *npr2* in 96hpf wild-type zebrafish embryos. Zebrafish embryos were captured at 96hpf and fixed with 4% PFA before wholemount in situ hybridization was performed to assess spatial expression of *nppc2*, *cnp3*, *nppc4* and *npr2* alongside sense probe controls. Images were captured at 20x (**A,B**) and 5x magnification (**C-F**) using bright field microscopy on an Axiovert 135 inverted microscope (Carl Zeiss Ltd, Cambridge, UK) and DC500 colour camera. *In situ* hybridization was performed using 12-15 embryos for each RNA probe and images are representative of the staining observed in multiple embryos. (PPH – presumptive pituitary and hypothalamus; F – forebrain; M – midbrain; H - hindbrain).

ZF nppcl CDS:

5' ATGCTGTGCTCATCTGTGTTTGTGGTGCTTCTTATCCTTTTGGCCAATCCCGTGCCGGGCAGCACCCGAGCCCTTCACACCTCTGACAAAGCCATGCAGGTCATTGAACAGTTCCTTGAGCGA

TACAATGACCTTTTAACCCTTGATGATCTTGAAAACCTCACAAGTGACCAATCGGAAGAGCCCGTGCAGACCTTCAGCTCTGGGCTAAAGGTTGCAGAAT**3'** TACGACACGAGTAGACACAAACA

CCACGAAGAATAGGAAAACCGGTTAG**GGCACGGCCCGTCGTGGGCTCGG**GAAGTGTGGAGACTGTTTCGGTACGTCCAGTAACTTGTCAAGGAACTCGCTATGTTACTGGAAAATTGGGAACTAC

TAGAACTTTTGGAGTGTTCACTGGTTAGCCTTCTCGGGCACGTCTGGAAGTCGAGACCCGATTTCCAACGTCTTA**5'** ATCCCAAGTGGATTGACATACCGGTGCAGAATGAGAATGCCTGGCTCC

GGCTTTTAAAGGGGGCTTTGGCCAATCAGAAACGAGCTCCACCGGATCGGTTGCGGAGGGGGTGGAACCGAGGCTGTTTTGGCTTGAAACTGGACCGGATCGGCTCTATGAGTGGCCTCGGCTGC

TAA**3'** TAGGGTTCACCTAACTGTATGGCCACGTCTTACTCTTACGGACCGAGGCCGAAAATTTCCCCCGAAACCGGTTAGTCTTTGCTCGAGGTGGCCTAGCCAACGCCTCCCCCACCTTGGCTC

CGACAAAACCGAACTTTGACCTGGCCTAGCCGAGATACTCACCGGAGCCGACGATT

CRISPR Guide: GGCTCGGGTGCTGCCCGGCA**CGG**

```
Fwd oligonucleotide:     5'TAGGCTCGGGTGCTGCCCGGCA
Common sequence:         3'    GAGCCCACGACGGGCCGT
Rvs sequence:            3'    GAGCCCACGACGGGCCGTCAAA
Rvs oligonucleotide:     5'AAACTGCCGGGCAGCACCCGAG
```

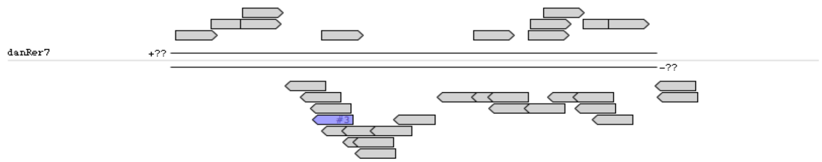

Supplemental Figure S3 Design of CRISPR/Cas9 gRNA for targeting zebrafish *nppcl*.
The whole genetic coding sequence for zebrafish *nppcl* (NM_001161341) was inserted into the CRISPR design software E---CRISP (http://www.e---crisp.org/E--- CRISP/designcrispr.html) and the MIT Optimized CRISPR design tool (http://crispr.mit.edu) using similar design parameters. The top scoring design sequences across from both design lists were then evaluated and the top scoring design present in both lists was selected. The target gRNA sequence is shown in the sequence above (blue) with the palindromic repeats at each end (red). Forward and reverse oligonucleotides designed are shown below along with the scoring output from the MIT Optimized CRISPR design tool.

guide #1 **quality score:** 99

guide sequence: GGCTCGGGTGCTGCCCGGCA CGG
on-target locus: chr16:+19266470
number of offtarget sites: 9 (4 are in genes)

top 20 genome-wide off-target sites ☐ show all exonic

sequence	score	mismatches	UCSC gene	locus
GGCTGGACTGCTGCCCAGCAGGG	0.4	4MMs [5:7:8:17]		chr24:+33047670
GCCTCGGCTGCTCCCCGCCACAG	0.1	4MMs [2:8:13:18]		chr18:+20112514
GTCTCAGGTGCTCCTCGGCAGAG	0.1	4MMs [2:6:13:15]		chr24:-1695503
GGAGCGGGTGCTGCCTGGTAAAG	0.1	4MMs [3:4:16:19]	NM_001045362	chr16:-25627614
CGCTCGGGTGCTGCGCAGTACAG	0.1	4MMs [1:15:17:19]	NM_001130643	chr2:+10814078
GGCTCGGCTGCAGCCCACCAGAG	0.1	4MMs [8:12:17:18]		chr11:-20201533
GGATCTGGTGCTGCTGGGCAGAG	0.0	4MMs [3:6:15:16]	NM_001044826	chr19:-33833284
GGCTCGTGTGCTTCTCTGCAAAG	0.0	4MMs [7:13:15:17]		chr9:+21957380
GGCTCTGGTGCTGCTGGGAAGAG	0.0	4MMs [6:15:16:19]		chr2:-35172825

**Gene**	**Accession Number**	**Forward primer** (5'-3'), **Reverse primer** (3'-5')	**Product Size** (including universal tag) (bp)	**Annealing Temperature** (^{o}C)
nppcl	NM_001161341	TCCTAGCAGGTGCCAAAAAC<br>GGTCCAGTTTCAAGCCAAAA	602	60.2
nppc4	XM_003201148	TTTGCTGGCTTGTGGACTTA<br>GAGAGGTTGTTAATGGAGTGG	379	60.4
nppc2	XM_001919491	TGGCTGCTTCATCATCTCTG<br>GTCCACCTTCATCCCAAAAC	332	56.4
cnp3	NM_001128741	GTGTCCTCGCTTCTGCTTTT<br>CACGGGTGGATTTAAAGAGG	557	60
npra	XM_002666526	ACCTCCTGTCCCGTATGGA<br>CCTGACACCACCATATACGC	352	60
npr1a	NM_001044937	CAGATGAGCAACACGGAGAA<br>TGATGCCAAAGCTGTAGACG	640	60
nprb	GU076493	GGGAGACCGTACAAGCAGAA<br>ACTCCATACGGGATGCTGTG	429	60
nprc	NM_001102673	ATTTTGCCGTGTTGAACTCC<br>ATCCCAGTCACCGCTGATAC	748	60.4
nppa	NM_198800	CCGGGGGACTAATTCTGAC<br>GAAGACCCTATGCGATCCAG	231	60
nppb	XM_002663224	AGTCGGCCTTCTCTTTCTCC<br>AGTTCTTCTTGGGACCTGAGC	365	60.9

Table S1: Custom oligonucleotide primers used for RT-PCR analyses and for construction of *in situ* hybridisation probes.

	Gene	Accession Number	Product Position (5'-3') (bp)	**Forward primer** (5'-3'), **Reverse primer** (3'-5')	**Product Size** (including universal tag) (bp)
1	*furinβ*	NM_001045109	2528-2627	AGGTGACACTATAGAATACTCCGGAGAGAACGAGTGTC<br>GTACGACTCACTATAGGGAAATGCTGGGGGATTTTCTCT	137
2	*nprb*	GU076493	2640-2746	AGGTGACACTATAGAATAACAGGTGGTGACATTGCTGA<br>GTACGACTCACTATAGGGAAGACCACCATGTACGCATCA	144
3	*BNP*	XM_002663224	321-434	AGGTGACACTATAGAATAGCAGCCCGATACTTACCTGA<br>GTACGACTCACTATAGGGACCGATTCTGTCCAGTTTGCT	151
4	*nppc2*	XM_001919491	778-898	AGGTGACACTATAGAATACTCCCTTGTATTGGCTCCTG<br>GTACGACTCACTATAGGGACTGTCTGAGGATGTCCAGCA	158
5	*nppc4*	XM_003201148	89-216	AGGTGACACTATAGAATACCCAGAATCAGCATCTCCAT<br>GTACGACTCACTATAGGGACTGAGGGACCTCTGTTCAGC	165
6	*18s*	NM_173234	122-256	AGGTGACACTATAGAATACCGCTATTAAGGGTGTTGGA<br>GTACGACTCACTATAGGGAGGCGAGGGTTCTGCATAATA	172
7	*nppa*	NM_198800	194-335	AGGTGACACTATAGAATAGGCATCAGAGAGAGCCGTAG<br>GTACGACTCACTATAGGGACTGTGTGTCAAATCCATCCG	179
8	*npr1a (both transcripts)*	NM_001044937	1389-1537	AGGTGACACTATAGAATAAAAAATGACAACCCTGCCTG<br>GTACGACTCACTATAGGGAAGCTGAGCGGTCAACTCATT	186
9	*eef1a1l1*	NM_131263	1113-1268	AGGTGACACTATAGAATACTGAACCACCCTGGTCAGAT<br>GTACGACTCACTATAGGGAATCTCCGGATTTGAGAGCCT	193
10	*nppcl*	NM_001161341	91-253	AGGTGACACTATAGAATAACACAGGATGGAGCAGGACT<br>GTACGACTCACTATAGGGACACCACTAATGCGAAGGGAT	200
11	*nprc (both transcripts)*	NM_001102673	2408-2578	AGGTGACACTATAGAATAGCAAGCCTTCCTGTATCCAA<br>GTACGACTCACTATAGGGAAGCGTCTGTACATGGCAGTG	208
12	*furinα*	NM_001045106	2081-2258	AGGTGACACTATAGAATAATCAAAAACGTGGCTGGAAC<br>GTACGACTCACTATAGGGAAGAAGCCGGGGTTACACTCT	215
13	*cnp3*	NM_001128741	157-342	AGGTGACACTATAGAATAGTGAACAAGCCGTTTGAGGA<br>GTACGACTCACTATAGGGAGCGCTTTGATGTGGACAGTA	223
14	*corin*	XM_005174157	2470-2664	AGGTGACACTATAGAATACTCCTGTTGTGAGACGCAAA<br>GTACGACTCACTATAGGGAGGCCTCCCAGAATTCTCTTC	232
15	*npra*	XM_002666526	127-331	AGGTGACACTATAGAATACCGTACCAGGTTGTGGACTT<br>GTACGACTCACTATAGGGAGGATCCTGAAGGTTTTGCAG	242

Table S2: Custom oligonucleotide primers used for multiplex RT-qPCR analyses

Gene expression	Exogenous CNP treatment	CRISPR *nppcl*-treatment
nppcl	Decreased	Decreased
nppc2	No change	Increased
cnp3	No change	No change
nppc4	Decreased/Increased	Increased
nppa	Increased	Decreased
bnp	No change	Increased
corin	Decreased/Increased	No change
furin a	Decreased	Decreased
furin b	No change	No change
npra	Increased/Decreased	Decreased
npr1a	Decreased/Increased	No change
nprb	Decreased	No change
nprc	No change	Increased

Morphometric analyses	Exogenous CNP treatment	CRISPR *nppcl*-treatment
Head width	Decreased	Decreased
Body length	Decreased	Decreased
Eye diameter	Decreased	Decreased

Table S3. Summary of gene expression and morphometric phenotypes in exogenous CNP treated zebrafish embryos and CRISPR *nppcl*-treated zebrafish embryos.
